# Short-Time Hydrothermal Synthesis of CuBi_2_O_4_ Nanocolumn Arrays for Efficient Visible-Light Photocatalysis

**DOI:** 10.3390/nano9091257

**Published:** 2019-09-05

**Authors:** Yi Wang, Fang Cai, Pengran Guo, Yongqian Lei, Qiaoyue Xi, Fuxian Wang

**Affiliations:** 1 College of Petrochemical Technology, Lanzhou University of Technology, Lanzhou 730050, China; 2 Guangdong Provincial Key Laboratory of Emergency Test for Dangerous Chemicals, Guangdong Engineering Technology Research Center of On-line Monitoring of Water Environmental Pollution, Guangdong Institute of Analysis, Guangzhou 510070, China

**Keywords:** CuBi_2_O_4_, short-time, hydrothermal method, photocatalytic degradation, methylene blue

## Abstract

In this article, a short-time hydrothermal method is developed to prepare CuBi_2_O_4_ nanocolumn arrays. By using Bi(NO_3_)_3_·5H_2_O in acetic acid and Cu(NO_3_)_2_·3H_2_O in ethanol as precursor solutions, tetragonal CuBi_2_O_4_ with good visible light absorption can be fabricated within 0.5 h at 120 °C. Tetragonal structured CuBi_2_O_4_ can be formed after 15 min hydrothermal treatment, however it possesses poor visible light absorption and low photocatalytic activity. Extending the hydrothermal treatment duration to 0.5 h results in a significant improvement invisible light absorption of the tetragonal CuBi_2_O_4_. The CuBi_2_O_4_ obtained through 0.5 h hydrothermal synthesis shows a band gap of 1.75 eV and exhibits the highest photocatalytic performance among the CuBi_2_O_4_ prepared with various hydrothermal time. The removal rate of methylene blue by the 0.5 h CuBi_2_O_4_ reaches 91% under visible light irradiation for 0.5 h. This study proposes a novel strategy to prepare photoactive CuBi_2_O_4_ nanocolumn arrays within 0.5 h at a moderate temperature of 120 °C. The hydrothermal method provides a facile strategy for the fast synthesis of metal-oxide-based photocatalysts at mild reaction conditions.

## 1. Introduction

CuBi_2_O_4_, a typical p-type semiconductor material, has emerged as a promising photocatalytic material due to its narrow bandgap (1.5–1.8 eV) and high photovoltage [1,2,3]. Specifically, tetragonal CuBi_2_O_4_ possesses strong visible light absorption and exhibits great potential for photocatalytic degradation of organic pollutants [4,5,6,7,8]. Different methods have been applied to prepare a CuBi_2_O_4_ photocatalyst, including the solid state grinding method [9,10,11], sol–gel method [12,13], and co-precipitation method [14]. However, these conventional methods generally suffer from high energy consumption, long fabrication time, or complex preparation procedures. As a cost effective and facile method, hydrothermal synthesis is commonly used to prepare various metal oxides (e.g., CuBi_2_O_4_, and BiVO_4_) with uniform size distribution, high purity, and high crystallinity [5,15,16]. It does not require organometallic or toxic precursors for the preparation nanocrystalline materials. However, traditional hydrothermal methods usually require long preparation time (generally 12–36 h for metal oxides, including CuBi_2_O_4_), which greatly limits their practical application [6,8,16,17,18,19,20,21].

In this study, we have successfully prepared tetrahedral CuBi_2_O_4_ nanocolumn arrays through a rapid hydrothermal method at mild reaction conditions. Tetrahedral CuBi_2_O_4_ with a band gap of 1.75 eV can be obtained within 0.5 h hydrothermal treatment at a moderate temperature of 120 °C. The 0.5 h CuBi_2_O_4_ nanocolumn arrays show good visible light absorption and photocatalytic property. The hydrothermal recipe developed for the synthesis of photoactive CuBi_2_O_4_ may be extended for the fast preparation of other metal-oxide-based photocatalysts at moderate reaction temperatures.

## 2. Materials and Methods

### 2.1. Material Preparation

The CuBi_2_O_4_ nanocolumn arrays were prepared by the hydrothermal method. Typically, 0.9701 g Bi(NO_3_)_3_·5H_2_O (99.999% Alfa Aesar (China) Chemical Co. Ltd., Shanghai, China) was dissolved in 5 mL acetic acid, and 0.2416 g Cu(NO_3_)_2_·3H_2_O (99.8% Alfa Aesar (China) Chemical Co. Ltd., Shanghai, China) was dissolved in 25 mL ethyl alcohol [22,23]. Then, the two solutions were mixed and NaOH solution was added under magnetic stirring at room temperature until pH 14 was reached. Subsequently, the above mixture was transferred into a 100 mL Teflon-lined steel autoclave, and then maintained in an oven at 120 °C for 5 min, 10 min, 15 min, 0.5 h, 1 h, 2 h, 8 h, and 12 h, respectively (the samples were denoted as CBO-5 min, CBO-10 min, CBO-15 min, CBO-0.5 h, CBO-1 h, CBO-2 h, CBO-8 h, and CBO-12 h, correspondingly). The obtained products were washed with ethanol and distilled water three times respectively and then dried in a vacuum drying oven for 12 h at 80 °C.

### 2.2. Characterization

The crystal structure was analyzed by an X-ray diffractometer (XRD, Bruker D 8 Advance, Karlsruhe, Germany) using Cu kα 1 as the radiation source. The morphologies of the CBO were characterized using a scanning electron microscopy (SEM, HITACHI S-3700N, Tokyo, Japan). X-ray photoelectron spectroscopy (XPS, ESCALAB 250XI, Thermo Fisher Scientific, Waltham, MA, USA) was carried out to investigate the stoichiometry and chemical bonding states of the samples. The UV-vis absorption spectra of the samples were collected by a UV-2800 spectrophotometer (Shanghai, China).

### 2.3. Photocatalytic Activity Measurement

First, 40 mg of the photocatalyst was dispersed into 50 mL methylene blue dye solution (0.02 mM). Then, 0.05 mL H_2_O_2_ was added and stirred in the dark for 30 min to reach the adsorption equilibrium. A 300 W xenon lamp with a 420 nm cut filter was used as the light source. Then, 2 mL aliquots were extracted and centrifuged every 5 min. The concentration of the remaining methylene blue was then analyzed by measuring the absorption of the supernate at 664 nm (the maximum absorption wavelength of methylene blue) via a UV-vis spectrophotometer. The degradation rate was calculated by the following formula:

(1) $$\mathrm{Degradation}\;\mathrm{rate}\;\left(\%\right)=1-\frac{C}{C_{0}}$$

where C_0_ and C refer to the absorbance of the solution at 664 nm before and after the test.

## 3. Results and Discussion

### 3.1. Synthetic Procedures of CuBi_2_O_4_ Nanocolumn Arrays

**Figure 1:**
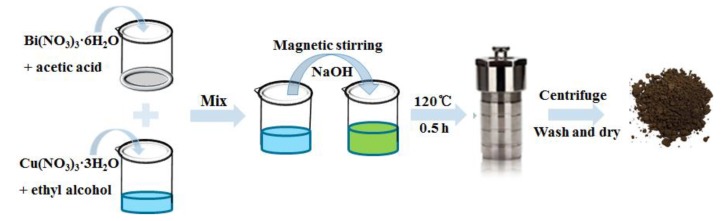
The preparation of CuBi_2_O_4_ via the hydrothermal treatment.

Different from traditional hydrothermal methods, the Bi(NO_3_)_3_·5H_2_O was dissolved in acetic acid to avoid the use of strong acid, and the Cu(NO_3_)_2_·3H_2_O was dissolved in ethanol instead of water in order to create high vapor pressure at moderate hydrothermal temperature. Those precursor solutions were previously used for the spray pyrolysis of CuBi_2_O_4_ thin films by Wang et al. [22]. It has been verified experimentally that both solutions can remain stable for at least 1 year at room temperature without stirring. As illustrated in Figure 1, a blue transparent solution was obtained when the colorless and transparent Bi(NO_3_)_3_ solution was mixed with the blue Cu(NO_3_)_2_ solution. When the NaOH solution was added dropwise to the mixture, the blue transparent solution first turned into a blue-white suspension, then a yellow-green suspension. After hydrothermal reaction for 0.5 h, a brownish black powder was finally obtained. The chemical reaction during the reaction can be expressed by the Formulas (2) to (5) [14]. The crystallization mechanism of CuBi_2_O_4_ particles can be described as a "dissolution-crystallization" process [14], during which the amorphous precipitate BiOOH and Cu(OH)_2_ are attacked by mineralizers in an alkaline environment to form ion aggregates. Subsequently, CuBi_2_O_4_ particles are produced through nucleation, precipitation, dehydration, and crystal growth in a supersaturated solution.

(2) $${\mathrm{Bi}}^{3+}+{3\mathrm{OH}}^{-}\to \mathrm{Bi}{\left(\mathrm{OH}\right)}_{3}$$

(3) $${\mathrm{Cu}}^{2+}+{2\mathrm{OH}}^{-}\to \mathrm{Cu}{\left(\mathrm{OH}\right)}_{2}$$

(4) $${\mathrm{Bi}(\mathrm{OH})}_{3}\to \mathrm{BiOOH}+H_{2}O$$

(5) $$2\mathrm{BiOOH}+{\mathrm{Cu}(\mathrm{OH})}_{2}\to {\mathrm{CuBi}}_{2}O_{4}$$

### 3.2. Characterization of CuBi_2_O_4_ Nanocolumn Arrays

**Figure 2:**
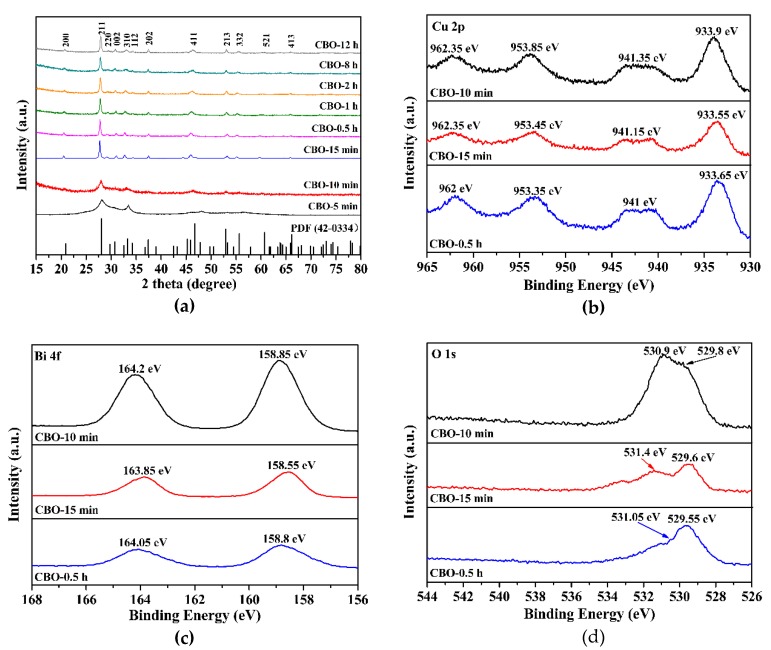
(**a**) XRD patterns of the CBO-5 min, CBO-10 min, CBO-15 min, CBO-0.5 h, CBO-1 h, CBO-2 h, CBO-8 h, CBO-12 h, and XPS high-resolution spectra of CBO-10 min, CBO-15 min, and CBO-0.5 h samples: (**b**) Cu 2p, (**c**) Bi 4f, and (**d**) O 1s.

Figure 2a shows the XRD patterns of CuBi_2_O_4_ with different hydrothermal durations. The CBO-10 min showed only two broad peaks appeared at 20.6° and 32.9°, indicating that the crystal growth was not complete in the first 10 min. When the hydrothermal time was 15 min, the peaks at 20.6°, 27.7°, 29.3°, 30.8°, 32.9°, 34.2°, 37.3°, 46.1°, 53.0°, 55.1°, 59.7°, and 65.8° were obviously observed, which can be assigned to the (200), (211), (220), (002), (310), (112), (202), (411), (213), (332), (521), and (413) lattice planes of tetragonal CuBi_2_O_4_ (JCPDF 42-0334), respectively. All the reflection peaks can be indexed to the tetragonal CuBi_2_O_4_ and no other impurities were found [23,24,25]. For the samples obtained under the hydrothermal times longer than 15 min, similar XRD patterns were obtained. It can be obviously noticed that the peak width at half-height of the CBO-10 min was wider than those with longer hydrothermal times, indicating small crystal size at the beginning of the crystal growth. To further investigate the surface chemical state of CuBi_2_O_4_, the CBO-10 min, CBO-15 min, and CBO-0.5 h samples were analyzed by XPS. The full XPS spectra of CBO samples displayed in Figure S1a confirmed the existence of Cu, Bi, and O elements. The C 1s high-resolution spectra of different samples were shown in Figure S1b. The binding energies were calibrated using the C 1s peak at 284.8 eV. It was observed that the three samples displayed similar elemental characteristic peaks. As shown in Figure 2b, the Cu^2+^peaks of the CBO-0.5 h shifted to lower binding energy of 933.65 eV and 953.35 eV, which corresponded to the Cu 2p_3/2_ and Cu 2p_1/2_ binding energies of Cu^2+^, respectively. Two shake-up satellites appeared at about 962 eV and 941 eV [1,2,26,27]. The high-resolution Bi 4f spectrum of pristine CBO-0.5 h in Figure 2c can be divided into two main peaks at 164.05 eV and 158.8 eV, which originated from Bi 4f_5/2_ and Bi 4f_7/2_ binding energies of Bi^3+^, respectively. The O 1s spectrum of CBO-0.5 was shown in Figure 2d, where the peak at 529.55 eV can be ascribed to the lattice oxygen [4,28,29]. As for the CBO-10 min and CBO-15 min samples, new peaks centered at about 530.9 eV and 531.4 eV in the XPS spectra of O 1s emerged (Figure 2d), which may represent the presence of subsurface oxygen vacancy defects [30].

**Figure 3:**
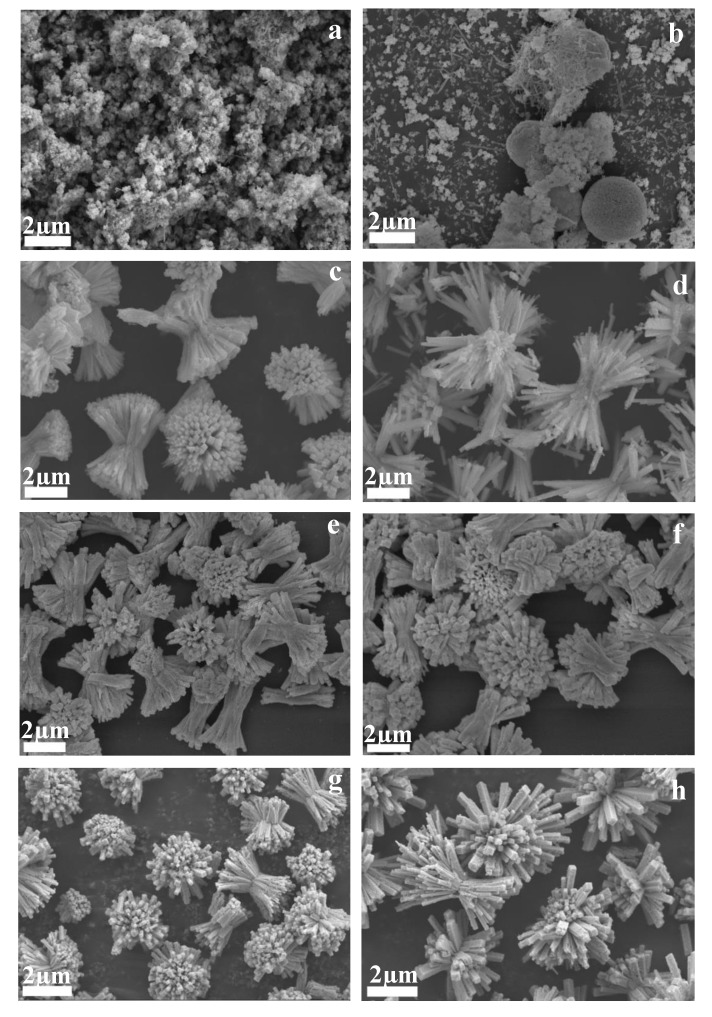
SEM images of CuBi_2_O_4_ prepared with the hydrothermal time of (**a**) 5 min, (**b**) 10 min, (**c**) 15 min, (**d**) 0.5 h, (**e**) 1 h, (**f**) 2 h, (**g**) 8 h, and (**h**) 12 h.

**Scheme 1:**
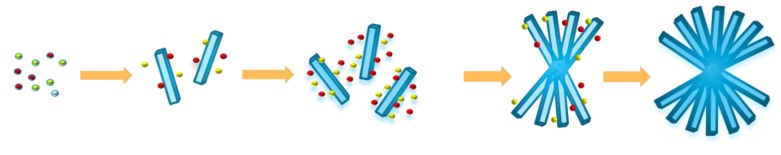
Schematic illustration for the preparation of CuBi_2_O_4_ nanocolumn arrays via the short-time hydrothermal synthesis.

To investigate the morphologies of CuBi_2_O_4_ with different hydrothermal time, SEM was conducted. When the hydrothermal time was only 5 min (Figure 3a), no obvious appearance was found except for some aggregated nanoparticles. As the hydrothermal time increased to 10 min (Figure 3b), some spherical particles were formed, which were dispersed and arranged in a random manner. After 15 min hydrothermal treatment (Figure 3c), hierarchical CuBi_2_O_4_ nanocolumn arrays were created. This is consistent with the XRD results in Figure 2d, where the CBO-5 min and CBO-10 min were found to be amorphous while the CBO-15 min showed well defined tetragonal structure. When the hydrothermal time increased from 0.5 h to 12 h, the hierarchical microstructures continued to grow. As shown in Figure 3d–f, the size of the nanorods increased and they became closer to each other, but in general the CuBi_2_O_4_ still appeared as nanorod arrays without substantial change in morphology. The proposed fabrication mechanism of CuBi_2_O_4_ nanocolumn arrays via the rapid hydrothermal synthesis route was illustrated in Scheme 1. Initially, the CuBi_2_O_4_ cores were created, based on which the nanorods started to grow. Then, the nanocolumns were obtained with the continuous generation and growth of the nanorods. Finally, the nanocolumn arrays were fabricated through the self-assembling of the nanocolumns. The repaid nucleation and high crystal growth rate of CuBi_2_O_4_ greatly shortened the hydrothermal reaction time and reduce the energy consumption.

### 3.3. Optical Absorption Properties

**Figure 4:**
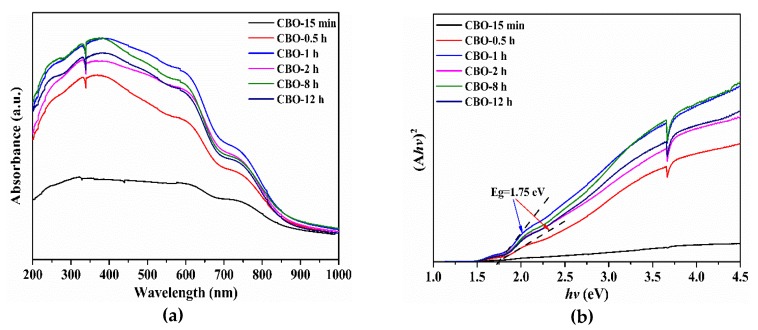
(**a**) UV-vis spectra and (**b**) plots of (Ahv)^2^ versus photon energy (hv) for the band gap energies of CBO-0.5 h, CBO-1 h, CBO-2 h, CBO-8 h, and CBO-12 h.

UV-vis diffuse reflectance spectra of CuBi_2_O_4_ with different hydrothermal time were investigated and displayed in Figure 4a. Tauc plots were applied to determine the band energy of different CuBi_2_O_4_ samples, as illustrated in Figure 4b. The CBO-15 min showed poor visible light absorption and its exact band gap could hardly be determined from the Tauc plot. In comparison with the CBO-15 min, the samples with longer hydrothermal time exhibited significantly enhanced visible light absorption. The CBO-0.5 h sample showed a direct band gap of 1.75 eV, which lies in the band gap range of CuBi_2_O_4_ as previously reported [22,23,28,31]. Further hydrothermal treatment for more than 1 h did not lead to improvement in visible light absorption.

### 3.4. Photocatalytic Activity of CuBi_2_O_4_

**Figure 5:**
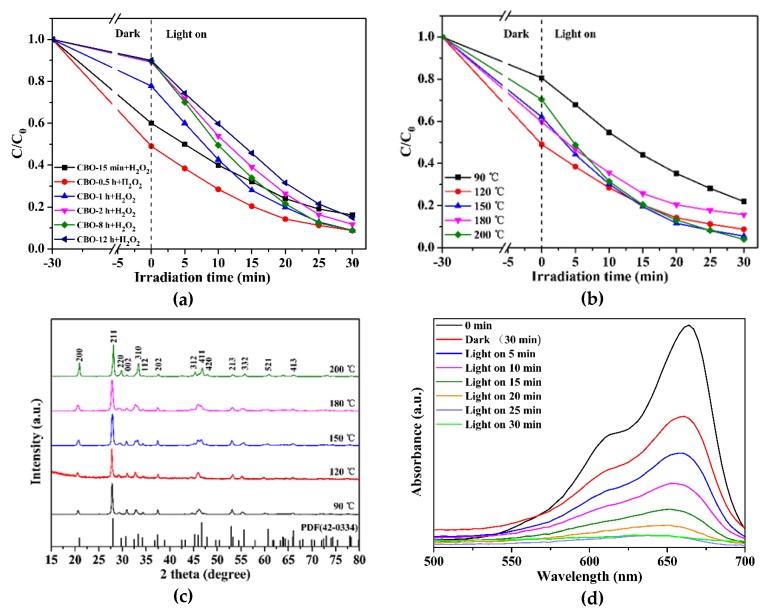
Photodegradation of methylene blue (MB) over as-prepared CuBi_2_O_4_ (**a**) with different hydrothermal time and (**b**) treated under different hydrothermal temperature (90 °C, 120 °C, 150 °C, 180 °C, and 200 °C) with the presence of 0.05 mL H_2_O_2_ under visible irradiation (λ ˃ 420 nm); (**c**) XRD patterns of CuBi_2_O_4_ treated under different hydrothermal temperature; and (**d**) the UV-vis spectra of MB degradation in the presence of CBO-0.5 h.

As showed previously in the XRD results, amorphous CuBi_2_O_4_ was obtained when the hydrothermal treatment time was shorter than 15 min. Therefore, the CuBi_2_O_4_ samples with treatment time over 15 min were selected to evaluate the photocatalytic performance via the degradation of methylene blue under visible light. As shown in Figure 5a, the CBO-15 min sample showed low photocatalytic performance, which can be ascribed to its poor visible light absorption (Figure 5a). CBO-0.5 h showed significantly higher photocatalytic activity than CBO-15 min, which can be explained by its good visible light absorption as shown in Figure 4. Among all the samples with different hydrothermal treatment durations, the CBO-0.5 h exhibited the highest photocatalytic performance, of which a removal rate of 91% was achieved within 30 min in the presence of a trace amount of H_2_O_2_ (about 0.05 mL in 50 mL solution). The photocatalytic degradation efficiency of the CBO-0.5 h is comparable to the previously reported CuBi_2_O_4_ prepared by traditional method with much longer hydrothermal treatment duration [6]. Further extending the hydrothermal time to 1 h or longer did not result in significant change in the photocatalytic performance. In general, photocatalysts with high specific surface areas and large pore volumes are beneficial for the enhancement of photocatalytic performance. To elucidate the effect of specific area, BET measurements were carried out. Figure S3 showed the nitrogen adsorption–desorption isotherms of CuBi_2_O_4_ with different hydrothermal time. It can be seen that all the samples had isotherms of type IV, indicating the presence of mesopores (2–50 nm). The isotherms exhibited H3 hysteresis loops at a high relative pressure range from 0.8 to 1.0, suggesting the presence of slit-like pores. As shown in Table S1, no significant difference in BET surface areas and pore volumes was found among the CBO samples with different hydrothermal time, therefore the difference in degradation rate should not be attributed to the specific surface area of samples. In addition, the optimization experiments of sodium hydroxide concentration showed that the concentration of the sodium hydroxide had no significant effect on the structure of the CuBi_2_O_4_ and 6.5 M sodium hydroxide was used in this study (Figure S2).

The photocatalytic performance of CuBi_2_O_4_ samples with different hydrothermal temperatures was also investigated. As shown in Figure 5b, CuBi_2_O_4_ sample with the hydrothermal temperature of 120 °C could achieve comparable photocatalytic performance as the one treated at a much higher temperature of 200 °C. When the temperature exceeded 120 °C, the (312) and (420) peaks appeared, nevertheless the emerged crystal planes of (312) and (420) had no promoting effect on the photocatalytic activity. From an energetic point of view, hydrothermal treatment at low temperature and low pressure is highly expected. Therefore, 120 °C was selected as a suitable hydrothermal temperature in this study. Eventually, we successively prepared the CuBi_2_O_4_ nanocolumn arrays via a facile hydrothermal method with much shorter reaction time at lower temperature compared to the traditional hydrothermal synthesis routes [4,7,14,25,30,32,33]. The time-dependent UV-vis absorption spectra of methylene blue (MB) under light irradiation for CBO-0.5 h at the hydrothermal temperature of 120 °C were presented in Figure 5d. The intensity of the absorption peak gradually decreased and eventually diminished as the irradiation time increased from 0 to 30 min.

### 3.5. Stability of the CuBi_2_O_4_ Nanocolumn Arrays

**Figure 6:**
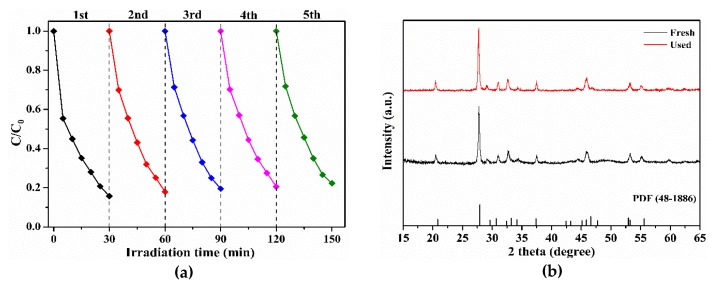
(**a**) Cycling runs for the photocatalytic MB degradation in the presence of CBO-0.5 h; and (**b**) XRD patterns of CuBi_2_O_4_ before and after cycle experiment.

The reusability of the CuBi_2_O_4_ samples was tested by repeating the photodegradation experiment under visible light irradiation. After each cycle of the photocatalysis experiment, the dye solution was adjusted to the initial concentration and the separated photocatalysts were washed and used again. As illustrated in Figure 6a, the decomposition rate of MB by the CBO-0.5 h slightly decreased by only 6.6% after five cycles of experiments, demonstrating the high stability of the as-prepared CuBi_2_O_4_ photocatalyst. We note that the decrease may be partially due to the loss of the sample powder during the separation and cleaning processes. The XRD patterns before and after 5 cycles of photodegradation experiments (Figure 6b) showed that the CBO-0.5 h maintained the tetragonal structure. 

## 4. Conclusions

In this study, we developed a facile hydrothermal method for the fast preparation of tetragonal CuBi_2_O_4_ nanorod arrays with good light absorption and high photocatalytic performance. The use of Bi(NO_3_)_3_·5H_2_O in acetic acid and Cu(NO_3_)_2_·3H_2_O in ethanol as precursor solutions allowed for the rapid hydrothermal synthesis of CuBi_2_O_4_ nanorod arrays at mild reaction conditions. Amorphous CuBi_2_O_4_ was obtained when the hydrothermal treatment time was shorter than 15 min. Tetragonal structured CuBi_2_O_4_ can be achieved when the hydrothermal treatment time reached 15 min, whereas the CBO-15 min presented poor visible light absorption and low photocatalytic performance. Extending the hydrothermal treatment duration to 0.5 h resulted in significantly improvement in visible light absorption and photocatalytic activity. Further prolonging the hydrothermal time to 1 h or even longer did not result in significant change in the photocatalytic performance. The CuBi_2_O_4_ with a hydrothermal treatment time of 0.5 h exhibited a degradation efficiency up to 91% for MB degradation, which is comparable to the CuBi_2_O_4_ prepared by traditional methods that commonly consume 12–36 h. Our hydrothermal method provides new insights for the rapid preparation of metal-oxide-based photocatalysts at moderate temperature and pressure.

## Supplementary Materials

The following are available online at https://www.mdpi.com/2079-4991/9/9/1257/s1, Figure S1: (a) XPS survey spectrum and (b) C 1s high-resolution spectra of the CBO-10 min, CBO-15 min, CBO-0.5 h; Figure S2: (a) XRD patterns of the under different concentration of NaOH (2.5 M, 4.5 M, 6.5 M, 8.5 M and 10.5 M), (b) Effects of CuBi_2_O_4_ with different concentration of NaOH (2.5 M, 4.5 M, 6.5 M, 8.5 M and 10.5 M) with the present of 0.05 mL H_2_O_2_ on the photocatalytic oxidation of MB under visible irradiation (λ ˃ 420 nm); Figure S3: N_2_ adsorption-desorption isotherms of the as-prepared samples; Table S1: The BET surface area, pore volume and average pore size of CBO-0.5 h, CBO-1 h, CBO-2 h, CBO-8 h, CBO-12 h.
