# Supplementary material for: Short-Time Hydrothermal Synthesis of CuBi2O4 Nanocolumn Arrays for Efficient Visible-Light Photocatalysis
Source: Nanomaterials (Basel). 2019 Sep 5;9(9):1257. doi: 10.3390/nano9091257 (PMC6780588; doi:10.3390/nano9091257)
Supplement: Supplementary file 1 [file nanomaterials-09-01257-s001.pdf]

nanomaterials MDPI

Supplementary Information

Short-Time Hydrothermal Synthesis of $CuBi_2O_4$ Nanocolumn Arrays for Efficient Visible-Light Photocatalysis

Yi Wang [1], Fang Cai [1,2], Pengran Guo [2], Yongqian Lei [2], Qiaoyue Xi [2,*] and Fuxian Wang [2,*]

1 College of Petrochemical Technology, Lanzhou University of Technology, Lanzhou 730050, China
2 Guangdong Provincial Key Laboratory of Emergency Test for Dangerous Chemicals, Guangdong Engineering Technology Research Center of On-line Monitoring of Water Environmental Pollution, Guangdong Institute of Analysis, Guangzhou 510070, China
* Correspondence:Correspondence: xiqiaoyue@fenxi.com.cn (Q.X.); wangfuxian@fenxi.com.cn (F.W.); Tel.: +86-020-37656885

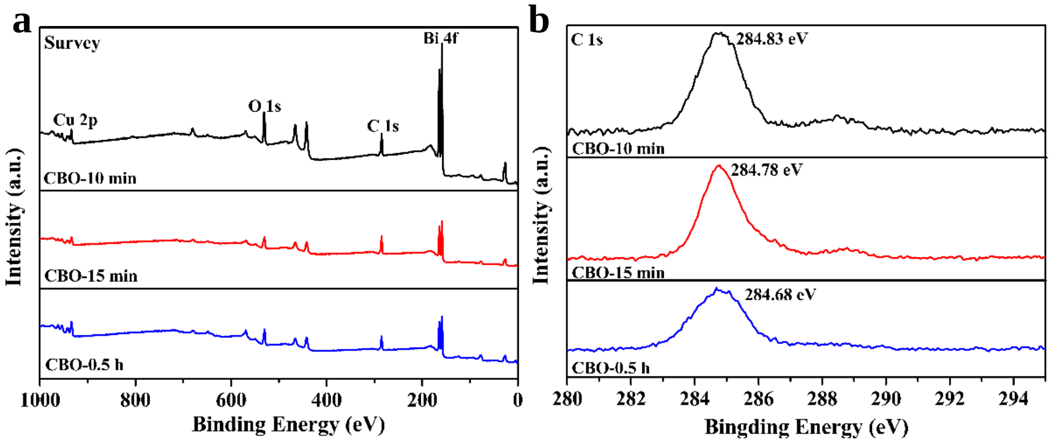

Figure S1. (a) XPS survey spectrum and **(b)** C 1s high-resolution spectra of the CBO-10 min, CBO-15 min, CBO-0.5 h.

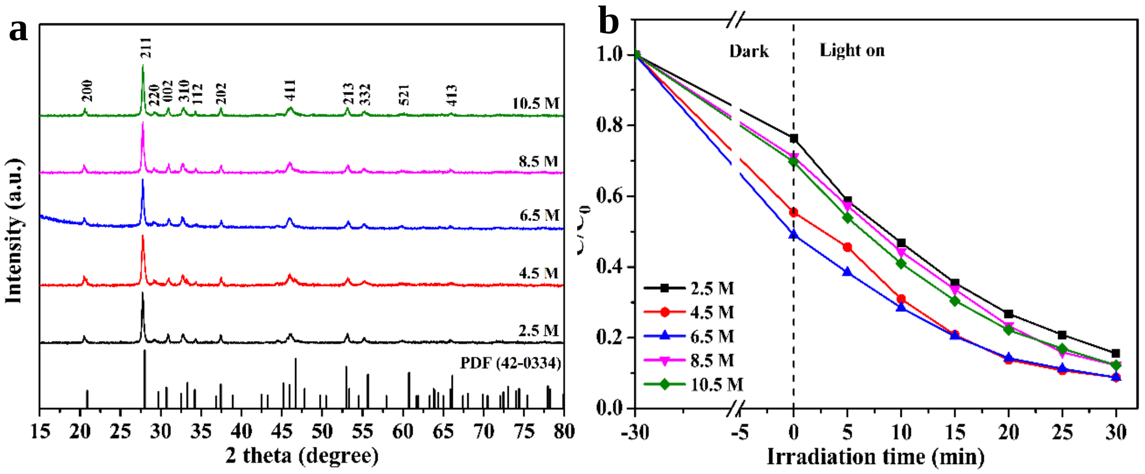

Figure S2. (**a**) XRD patterns of the under different concentration of NaOH (2.5 M, 4.5 M, 6.5 M, 8.5 M and 10.5 M).(**b**)Effects of $CuBi_2O_4$ with different concentration of NaOH (2.5 M, 4.5 M, 6.5 M, 8.5 M and 10.5 M) with the present of 0.05 mL H_2O_2 on the photocatalytic oxidation of MB under visible irradiation (λ > 420 nm).

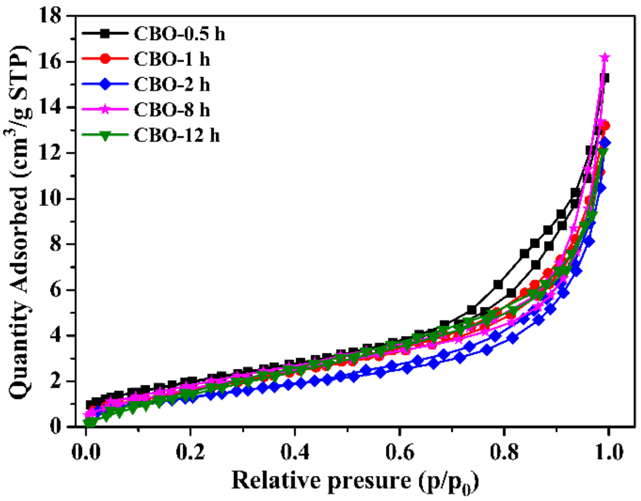

Figure S3. N_2 adsorption-desorption isotherms of the as-prepared samples.

Table 1. The BET surface area, pore volume and average pore size of CBO-0.5 h, CBO-1 h, CBO-2 h, CBO-8 h, CBO-12 h.

Samples	CBO-0.5	CBO-1	CBO-2	CBO-8	CBO-12
BET (m^2/g)	7.788	6.940	5.339	7.385	7.233
Pore volume (cm^3/g)	0.023	0.019	0.018	0.023	0.019
Average pore size (nm)	9.367	8.554	10.421	9.867	6.922
